# Unmasked insulinoma occasioned by severe hypoglycemic coma immediately postpartum: a case report

**DOI:** 10.1186/s12902-023-01415-1

**Published:** 2023-08-10

**Authors:** Kiyoshi Matsumoto, Miyu Watanabe, Ken Takao, Hirokazu Takahashi, Hisashi Daido, Toshiro Shibata, Tokuyuki Hirose, Takehiro Kato, Masami Mizuno, Takuo Hirota, Tetsuya Suwa, Yukio Horikawa, Takaaki Murakami, Daisuke Yabe

**Affiliations:** 1 Department of Diabetes, Endocrinology and Metabolism and Department of Rheumatology and Clinical Immunology, Gifu University Graduate School of Medicine, Gifu, 501-1194 Japan; 2 Department of Diabetes and Endocrinology, Gifu Prefectural General Medical Center, Gifu, Japan; 3 Department of Internal Medicine, Japanese Red Cross Takayama Hospital, Takayama, Japan; 4 Department of Diabetes, Endocrinology and Nutrition, Kyoto University Graduate School of Medicine, Kyoto, Japan; 5 Yutaka Seino Distinguished Center for Diabetes Research, Kansai Electric Power Medical Research Institute, Kobe, Japan; 6 Preemptive Food Research Center, Gifu University Institute for Advanced Study, Gifu, Japan; 7 Center for One Medicine Innovative Translational Research, Gifu University, Gifu, Japan

**Keywords:** Insulinoma, Postpartum hypoglycemic coma, SACST

## Abstract

**Background:**

Insulinoma in women during pregnancy and postpartum is very rare; approximately 65% of cases are diagnosed early in pregnancy and ~ 35% immediately after delivery, few being found in middle or late pregnancy, likely due to increased insulin resistance seen after early-stage pregnancy. We successfully treated a case of insulinoma in which severe hypoglycemic coma immediately after delivery occasioned detailed investigation and diagnosis.

**Case presentation:**

Our patient experienced hypoglycemic coma in the 3^rd^ month of pregnancy (initially considered due to her hyperemesis gravidarum) that improved spontaneously during the gestational period. No abnormalities of plasma glucose or body weight were found in regular checkups during her pregnancy; however, recurrence of hypoglycemic coma after delivery led us to suspect insulinoma. While contrast enhanced computer tomography and endoscopic ultrasonography (EUS) initially failed to detect a tumor in the pancreas, selective arterial calcium stimulation test revealed an insulin-secreting tumor localized in the pancreatic body. She then underwent spleen-preserving distal pancreatectomy; a 10-mm tumor positive for chromogranin A, synaptophysin and insulin was identified.

**Conclusions:**

Although pregnancy can mask insulinoma-associated symptoms and make diagnosis challenging, hypoglycemic episodes during early pregnancy, which were observed in this case, are suggestive of insulinoma. Importantly, in this case, accurate preoperative localization of the tumor enabled prompt curative surgery after delivery. Thus, clinical vigilance for the occurrence of insulinoma and its localization is appropriate for pregnant women suffering severe hypoglycemia.

## Background

Insulinoma, the most common functional neuroendocrine tumor in the pancreas, is nevertheless a rare neuroendocrine tumor that is derived from pancreatic β-cells [1]. The disease is characterized by hyperinsulinemia independent of glucose level, fasting hypoglycemia and body weight gain. Whipple’s triad (three markers: symptoms of hypoglycemia, low plasma glucose level and relief of symptoms in response to raising glucose elevation) indicates insulinoma. Early diagnosis is required; delay leads to weight gain, memory impairment and decreased intelligence and can readily progress to coma or death [2–4]. It has been demonstrated recently that continuous subcutaneous glucose measurement and intermittently scanned glucose monitoring (isCGM) are useful for early diagnosis of the disease [5, 6], although the 72-h fasting test remains the gold standard of diagnosis [7]. Treatment of insulinoma is primarily by surgical resection; it is therefore important to localize the tumor(s) to ascertain the area of the pancreas to be resected [8–10]. It has been shown in 237 patients that the accuracy of contrast enhanced-computer tomography (CE-CT), endoscopic ultrasonography (EUS) and selective arterial calcium stimulation test (SACST) is 55%, 75% and 93%, respectively [11].

Although insulinoma is clinically rare, it has been reported in women during pregnancy and immediately postpartum. In these cases, approximately 65% were diagnosed early in pregnancy and the remainder immediately postpartum, few cases being found in middle or late pregnancy, likely because insulin resistance is increased after the early stage of pregnancy [12–16]. Importantly, in approximately 75% of the cases, loss of consciousness ranging from disorientation to syncope are the only symptoms of insulinoma [14], which suggests that the prevalence of masked hypoglycemia may be high, impeding prompt diagnosis.

We report here a case of insulinoma in which severe hypoglycemic coma immediately postpartum occasioned detailed investigation and diagnosis of the disease. This case highlights the difficulties in diagnosing insulinoma during pregnancy as well as the importance of clinical vigilance for insulinoma as a cause of hypoglycemia during pregnancy.

## Case presentation

**Fig. 1:**
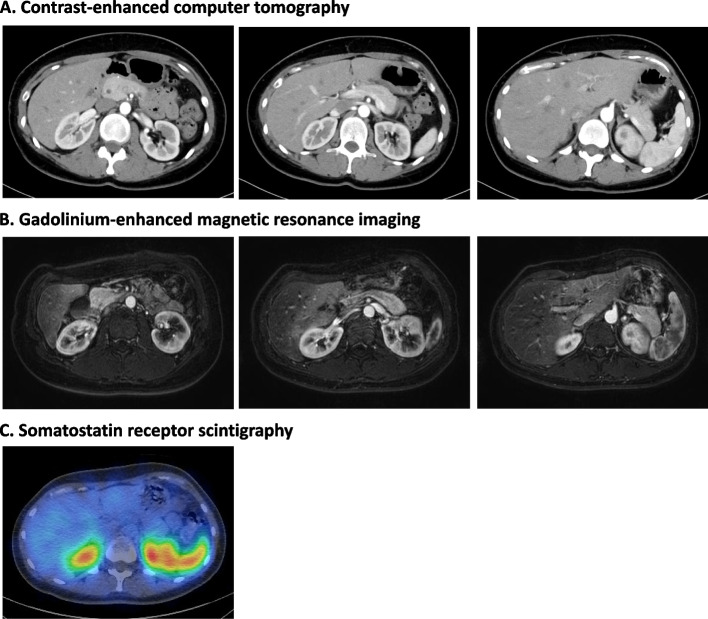
Imaging analyses of the pancreas of the case. **A** Contrast-enhanced computer tomography. **B** Gadolinium-enhanced magnetic resonance imaging. **C** Somatostatin receptor scintigraphy

**Fig. 2:**
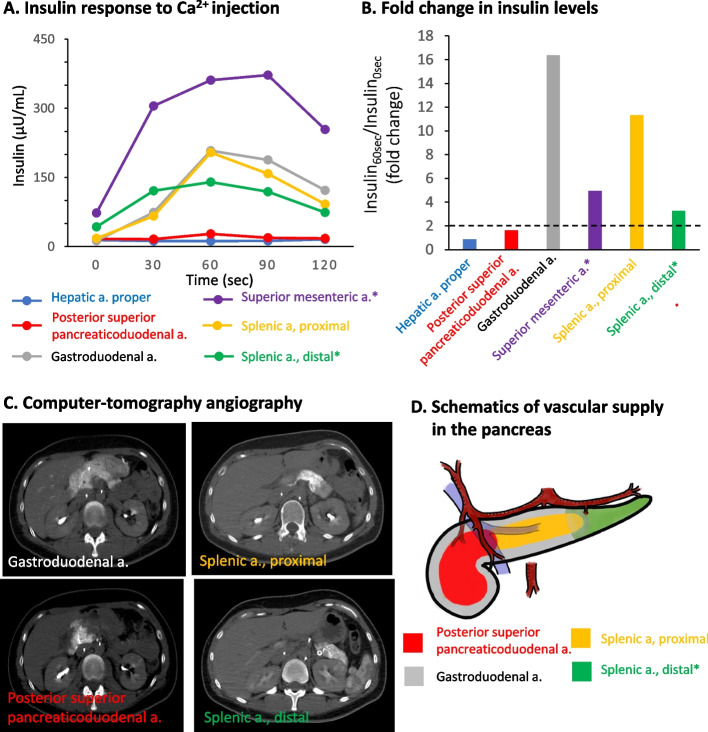
Selective arterial calcium stimulation test in the case. **A** Selective arterial calcium stimulation test (SACST) was performed after confirming vascular supply of the pancreas by computer tomography angiography. Insulin levels are plotted before and after injection of calcium gluconate into indicated arteries. As she experienced hypoglycemia (plasma glucose level 30 mg/dL or less) during SACST, 50% glucose solution was given intravenously before injection of calcium gluconate into the arteries denoted by asterisks (i.e., the superior mesenteric artery and the distal splenic artery). **B** Fold changes in insulin levels before and 60 s after injection of calcium gluconate (Insulin 60 s/Insulin 0 s) are shown for each indicated artery. Note that fold changes for the superior mesenteric artery and the distal splenic artery are relatively low, presumably because insulin levels before the injection of calcium gluconate were relatively high and the patient had experienced hypoglycemia and received intravenous injection of 50% glucose solution. **C** Computer tomography angiography visualization of the vascular supply of the pancreas. Note that injection of contrast medium into the hepatic artery proper and the superior mesenteric artery did not result in significant contrast effects in the pancreas. **D** Schematics of the vascular supply in the pancreas. Note that the gastroduodenal artery feeds a relatively large area of the pancreas (head and body)

**Fig. 3:**
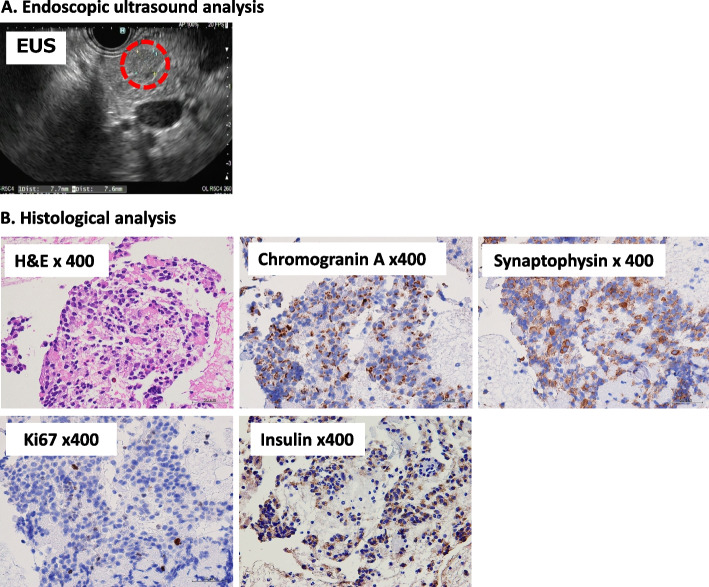
Endoscopic ultrasound and histological analyses of specimen aspirated from the pancreatic tumor. **A** Endoscopic ultrasound image of a 7 mm-hypoechoic tumor of the pancreatic body. **B** Histological findings of specimen aspirated from the pancreatic tumor. Sections of the aspirated specimen were stained by hematoxylin and eosin as well as by chromogranin A, synaptophysin, insulin and Ki67 (Magnification × 400). Tumor cells were positive for chromogranin A and synaptophysin, which is typical of neuroendocrine tumors. The proliferation index, assessed by Ki67 immunostaining, was < 2.0%. Tumor cells were positive for insulin but not for glucagon, gastrin, or somatostatin (data not shown)

**Fig. 4:**
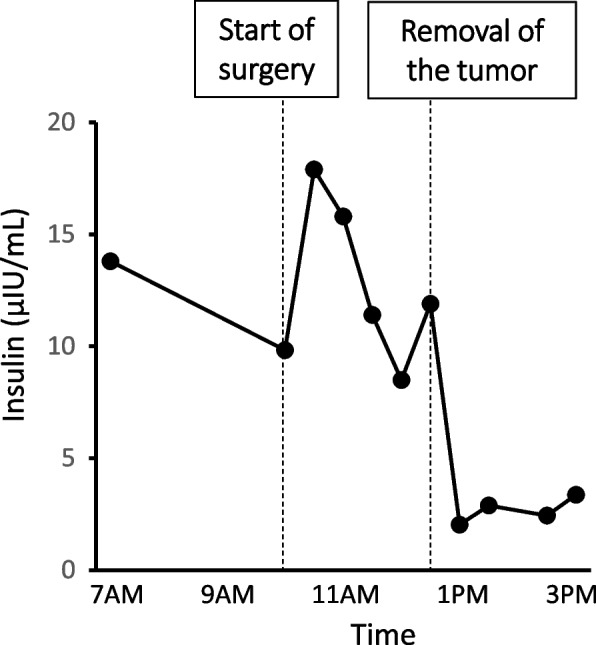
Change of insulin levels before and after resection of the pancreatic tumor. Insulin levels are plotted before and after resection of the pancreatic tumor. Intraoperative ultrasound examination was performed to confirm the localization of a 7 mm-sized hard and palpable tumor in the tail of the pancreas near the body of the pancreas. After removal of the tumor, insulin levels were drastically declined

**Fig. 5:**
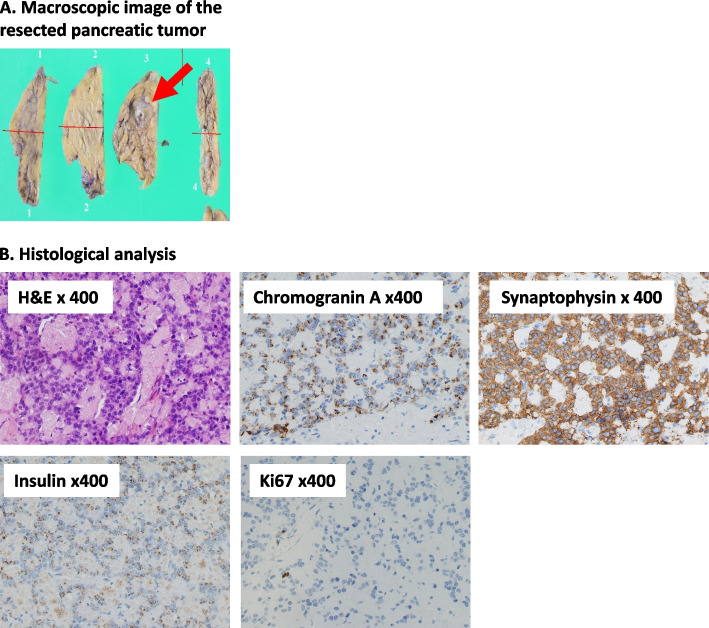
Histological analyses of the resected pancreatic tumor. **A** Macroscopic image of the resected tumor (Arrow, 10 × 8 mm, well-defined yellowish mass). **B** Histological findings of the resected pancreatic tumor. Sections of the tumor were stained by hematoxylin and eosin (H&E) as well as chromogranin A, synaptophysin, insulin and Ki67 (Magnification × 400). Tumor cells were positive for chromogranin A, synaptophysin and insulin, and the proliferation index, assessed by Ki67 immunostaining, was < 2.0% (NET, G1, according to WHO 2019). Tumor cells were negative for glucagon, gastrin, and somatostatin (data not shown)

The patient was a 40-year-old woman. She has no family history of endocrine disease, including hyperparathyroidism, pituitary adenoma and pancreatic gastrointestinal endocrine tumors. She was married at age 31, started fertility treatment at age 35 and became pregnant at age 39 by in vitro fertilization. During the 3^rd^ month of pregnancy, she had difficulty consuming meals due to hyperemesis gravidarum; she was treated in the emergency room of her nearby hospital after she was found unconscious at home. Upon arrival at the hospital, her plasma glucose level was 36 mg/dL. Because her symptoms disappeared soon after an intravenous glucose infusion, she was discharged from the emergency room without further investigation of the cause of the hypoglycemic episode. Afterward, she had no hypoglycemic coma, abnormalities of plasma glucose or significant body weight gain in regular checkups during the pregnancy. No abnormalities were noted in the 75-g oral glucose tolerance test performed at week 33 of pregnancy (Plasma glucose levels: 0 min, 60 mg/dL, 60 min, 100 mg/dL and 120 min, 138 mg/dL). Because the baby was in breech position near the due date, she underwent caesarean section at week 38 of pregnancy and gave birth to a healthy child with body weight of 2,595 g. She had no complications associated with the caesarean section; she began eating meals 2 days after the operation. However, she was found unconscious in her home early in the morning 3 days after the operation. Her point-of-care blood glucose level was 16 mg/dL; her symptoms disappeared soon after an intravenous glucose infusion. She was then referred to an endocrinologist in the same hospital, who undertook further investigation of the hypoglycemic episode. isCGM revealed frequent nocturnal hypoglycemic episodes. She was negative for insulin autoantibodies and her cortisol level in the early morning was 38.2 μg/dL (normal range, 4.5–21.1 μg/dL), which excluded the possibilities of insulin autoimmune syndrome and adrenal insufficiency as the cause. She was then hospitalized and subjected to a 72-h fasting test. The test was stopped 7 h after beginning because of her low plasma glucose level (30 mg/dL). Despite the low plasma glucose, her insulin (7.30 μU/mL: normal range, < 18.7 μU/mL) and C-peptide (1.97 ng/mL: normal range, 0.61–2.09 ng/mL) levels were inappropriately high, and the blood glucose level was increased to 109 mg /dL at 20 min after an intravenous glucagon infusion, all of which findings are consistent with insulinoma. However, CE-CT and EUS failed to detect responsible lesions in the pancreas. Consequently, she started receiving diazoxide 50 mg t.i.d. orally, which reduced the frequency of the hypoglycemic episodes, but she had difficulty with the drug due to the development of lower leg edema. She was therefore referred to our institution for further evaluation and treatment. Upon admission, her BMI was 25.4 kg/m^2^ (height 153.2 cm; body weight 59.5 kg). She had no family history of diabetes or other endocrine disorders. Her serum insulin level (6.34 μU/mL) was relatively high compared to her plasma glucose level (67 mg/dL) (Table 1). No tumor was found in the pancreas by CE-CT, gadolinium enhanced magnetic-resonance imaging (Gd-MRI) or somatostatin receptor scintigraphy (Fig. 1). Thereafter, (SACST) in conjunction with computer tomography angiography, which visualizes the arteries feeding the pancreas, was performed to localize the responsible lesion after 3-day discontinuation of diazoxide. A significant increase in insulin level was observed, especially in the gastroduodenal and proximal splenic arteries, indicating localization of an insulin-secreting tumor in the pancreatic body and tail (Fig. 2). EUS was then performed on the pancreatic body and tail, revealing a 7-mm low-echoic mass in the pancreatic body (Fig. 3A). Histological analysis of a specimen aspirated from the mass found it to be an insulin-secreting neuroendocrine tumor (NET, G1, according to WHO 2019) (Fig. 3B). An open spleen-preserving distal pancreatectomy was then performed under intraoperative ultrasound guidance. The operating time was 4 h and 15 min, and the blood loss was 155 ml. Intraoperative insulin measurements showed a rapid decline in serum insulin levels after the resection, indicating successful removal of the tumor (Fig. 4). The insulin-secreting tumor was 10-mm in diameter and consisted microscopically of atypical cells positive for chromogranin A, synaptophysin and insulin but negative for glucagon and somatostatin. Both the mitotic and Ki-67 indices were low, and the tumor was classified as NET, G1, according to the WHO 2019 criteria (Fig. 5) [17]. There were periods of fasting and administration of antibiotics due to abdominal pain and effusion in the pancreatic stump, and she was discharged on the 22^nd^ post operative day. After the operation, the patient showed no further symptoms of hypoglycemia.

**Table 1:** Biochemistry and complete blood count upon hospitalization in our institution

**Biochemistry**				**Blood count coagulation**	
T-Bil	0.5 mg/dL	HbA1c	4.7%	WBC	3190 /μL
TP	6.0 g/dL	GLU	67 mg/dL	RBC	388 × 10^4^ /μL
ALB	3.6 g/dL	TG	67 mg/dL	Hb	11.0 g/dL
CK	52 U/L	HDL-C	72 mg/dL	Hct	33.2%
AST	14 U/L	LDL-C	98 mg/dL	PLT	14.9 × 10^4^ /μL
ALT	9 U/L	**Endocrine**		APTT	30.3秒
γ-GTP	8 U/L	TSH	2.46 mIU/mL	PT	> 120%
Cre	0.57 mg/dL	FT3	2.77 pg/mL	PT-INR	0.88
UA	4.9 mg/dL	FT4	1.03 ng/dL	**Urinalysis**	
BUN	16.1 mg/dL	ACTH	12.0 pg/mL	Specific gravity	1.022
Na	141 mEq/L	Cortisol	5.2 μg/dL	pH	7.0
K	3.7 mEq/L	Insulin	6.34μIU/mL	Protein	( ±)
Cl	109 mEq/L	**Autoantibodies**		Glucose	(-)
Ca	8.1 mEq/L	Anti-insulin Ab	< 0.4 U/mL	Ketone body	(-)
IP	2.8 mg/dL			Blood	(-)

*ALP* Alkaline phosphatase, *AST* Aspartate aminotransferase, *ALT* Alanine aminotransferase, *Hb* Hemoglobin, *Ht* Hematocrit, *γGTP* γ-glutamyltransferase, *LDH* Lactate dehydrogenase, *RBC* Red blood cells, *TP* Toral protein, *WBC* White blood cells, *ALB* Albumin, *T-Bil* Total bilirubin, *HDL-C* HDL-cholesterol, *LDL-C* LDL-cholesterol, *TSH* thyroid-stimulating hormone, *FT3* free triiodothyronine, *FT4* Free thyroxine, ACTH Adenocorticotropic hormone, *Anti-insulin Ab* Anti-insulin antibody

**Discussion** and** conclusions.**

In Japan, the prevalence and incidence of pancreatic neuroendocrine tumors are 2.69 and 1.27 per 100,000 people, respectively, and insulinoma accounts for 20.9%. It occurs in each age group, has an average age of 45 years and approximately 30% of cases occur in people in their 20 s to 40 s. The male-to-female ratio is 1:14, slightly more common in females [1, 18, 19].

Early diagnosis of insulinoma is often difficult because patients are unaware of hypoglycemic symptoms. It is even more difficult in pregnant women because hypoglycemic symptoms in the early stage of pregnancy may be mistaken for those of hyperemesis gravidarum, and insulinoma-related symptoms rarely occur after mid-pregnancy due to the enhancement of insulin resistance seen in pregnancy [20–22]. Retrospectively, it is likely that our case’s hypoglycemic episodes were caused by insulinoma rather than hyperemesis gravidarum as originally suspected when hypoglycemic coma first occurred in the 3^rd^ month of pregnancy. She showed no abnormalities in plasma glucose level or body weight in regular checkups during pregnancy, presumably due to pregnancy-associated enhancement of insulin resistance [20–22]; however, she experienced severe hypoglycemic coma immediately after delivery when insulin resistance was normalized [23]. This case highlights the difficulty in diagnosing insulinoma during pregnancy and the importance of clinical vigilance for severe postpartum hypoglycemic coma.

Surgical resection is currently recommended as the treatment for insulinoma, localization of the tumor(s) being critical. Approximately 90% of insulinomas begin and grow in the pancreas. Enucleation is recommended for insulinomas of diameter ≤ 20 mm but is not recommended if the distance between the tumor and the main pancreatic duct is < 3 mm, in order to prevent injury. Localization of the insulinoma was especially difficult in the current case because imaging analyses such as CE-CT, Gd-MRI and EUS initially failed to detect lesions in the pancreas. We employed CT angiography-assisted SACST to localize the tumor; their usefulness was reaffirmed [24]. CE-CT and Gd-MRI are especially challenging in pregnant women, and there are few other means to diagnose and localize insulinoma in them. In the future, non-invasive diagnostic methods such as Ex4 PET/CT may be available [25–27].

In conclusion, we report a case of insulinoma in which postpartum hypoglycemic coma prompted detailed examination and diagnosis of the disease; SACST permitted localization and successful resection of a small insulin-producing tumor in the pancreas. Clinical vigilance for insulinoma in pregnant women suffering severe hypoglycemia is therefore necessary.

## Data Availability

Clinical data from the corresponding author is available upon request.

## Abbreviations

EUS: Endoscopic ultrasonography
isCGM: Intermittently scanned glucose monitoring
CE-CT: Enhanced-computer tomography
SACST: Selective arterial calcium stimulation test
Gd-MRI: Gadolinium enhanced magnetic-resonance imaging
